# Differentially Expressed Genes and Their Clinical Significance in Ischaemic Stroke: An In-Silico Study

**DOI:** 10.21315/mjms2020.27.6.6

**Published:** 2020-12-29

**Authors:** Sandeep Appunni, Muni Rubens, Venkataraghavan Ramamoorthy, Hina Sharma, Anjani Kumar Singh, Vishnu Swarup, Himanshu Narayan Singh

**Affiliations:** 1 Government Medical College, Kozhikode, Kerala, India; 2 Miami Cancer Institute, Florida, USA; 3 University of Central Missouri, USA; 4 National Network of Depression Centers India Foundation, New Delhi, India; 5 Atma Ram Sanatan Dharma College, University of Delhi, New Delhi, India; 6 All India Institute of Medical Sciences, New Delhi, India; 7 Aix Marseille University, Marseille, France; 8 MTA Infotech, New Delhi, India

**Keywords:** ischaemic stroke, differential mRNA expression, gene set enrichment analysis, gene ontology, GPM6B, JunD

## Abstract

**Background:**

Ischaemic stroke (IS), a multifactorial neurological disorder, is mediated by interplay between genes and the environment and, thus, blood-based IS biomarkers are of significant clinical value. Therefore, this study aimed to find global differentially expressed genes (DEGs) in-silico, to identify key enriched genes via gene set enrichment analysis (GSEA) and to determine the clinical significance of these genes in IS.

**Methods:**

Microarray expression dataset GSE22255 was retrieved from the Gene Expression Omnibus (GEO) database. It includes messenger ribonucleic acid (mRNA) expression data for the peripheral blood mononuclear cells of 20 controls and 20 IS patients. The bioconductor-package ‘affy’ was used to calculate expression and a pairwise *t-*test was applied to screen DEGs (*P <* 0.01). Further, GSEA was used to determine the enrichment of DEGs specific to gene ontology (GO) annotations.

**Results:**

GSEA analysis revealed 21 genes to be significantly plausible gene markers, enriched in multiple pathways among all the DEGs (*n =* 881). Ten gene sets were found to be core enriched in specific GO annotations. JunD, NCX3 and fibroblast growth factor receptor 4 (FGFR4) were under-represented and glycoprotein M6-B (GPM6B*)* was persistently over-represented.

**Conclusion:**

The identified genes are either associated with the pathophysiology of IS or they affect post-IS neuronal regeneration, thereby influencing clinical outcome. These genes should, therefore, be evaluated for their utility as suitable markers for predicting IS in clinical scenarios.

## Introduction

Stroke is a focal or infrequently, a systemic, neurological impairment of sudden onset, which lasts for more than 24 h and is of vascular origin (1). The perfusion inadequacy characteristic of stroke is either due to a focal ischaemia or to a haemorrhage affecting cerebral blood flow (2–3). Stroke is commonly associated with modifiable risk factors, such as diabetes mellitus, high body mass index (BMI > 30 kg/m^2^), hypertension, cigarette smoking and carotid artery disease (4–5). In the United States, stroke is the fifth major cause of mortality, with nearly 800,000 cases and 120,000 deaths per year (6). The current management of ischaemic stroke (IS; from acute to chronic) emphasises acute intensive care interventional therapy (including thrombolytics), lifestyle changes, recurrence-preventing medication and physiotherapy measures for rehabilitation. The genetic aetiology for stroke and its risk factors is very complex, as stroke is usually a polygenic condition, meaning that multiple gene loci are affected. However, monogenic forms of stroke have also been reported and serve as models for understanding the influence of environmental or genetic factors on stroke occurrence (7).

Identifying characteristic genomic alterations associated with stroke prior to stroke occurrence can enable the implementation of better surveillance and treatment strategies to manage the at-risk population. Experimental, computer-aided and mathematical models have improved the current understanding of the alterations that occur in IS transcriptomics (8). Genome-wide gene expression studies and transcriptome analyses are platforms for comparatively analysing multiple genes at once. Such examinations reveal significant gene expression differences associated with a given disease. The differential expression of genes between the diseased and normal state has potential implications for diagnosis and therapeutic management. Thus, from the perspective of personalised and precision medicine, identifying and targeting key transcriptomic alterations are valuable tools for managing IS. Although in-silico analysis is a cumbersome data analysis method, it does provide a plausible roadmap for biologists. Gene set enrichment analysis (GSEA), in particular, may reveal important aspects of disease aetiology by identifying, pooling and functionally characterising significantly altered genes within the genome. Earlier studies have shown that genome-wide expression analysis permits the simultaneous identification of gene clusters that are altered in the diseased state (9–10). Simultaneously analysing gene ontology (GO) and the pathway enrichment associated with the altered gene set will provide further information about the molecular networks involved in the diseased state. Transcriptome analyses in animal model of IS have revealed differentially expressed messenger ribonucleic acid (mRNA) and micro ribonucleic acid (miRNA), presenting as clustered sets of unique markers that are pathologically expressed and correlate well with different disease stages (11).

Though diagnostic modalities for stroke have advanced, therapeutic strategies remain limited to thrombolytics, which has a narrow therapeutic window. The limited application of translational genomics into stroke management is keeping the scope of personalised medicine in its infancy. However, exploring the genetic pool will open a plethora of gene expression profiles, polymorphisms and chromosomal abnormalities, which will help practitioners identify the pathophysiological event well in advance and structure a more specific, targeted therapeutic approach. This study, therefore, aimed to investigate differential gene expression patterns obtained from the peripheral blood samples of IS patients. Subsequently, GSEA was performed with the dataset to identify key genomic alterations in specific molecular pathways, which could affect stroke occurrence and outcome.

## Methods

### Affymetrix Microarray Data

The mRNA microarray expression dataset GSE22255, which is based on the Affymetrix Human Genome U133 Plus 2.0 Array (Affymetrix, Inc., Santa Clara, CA, USA), was retrieved from the Gene Expression Omnibus (GEO). This dataset includes mRNA expression data from the peripheral blood mononuclear cells of 20 controls and 20 IS patients. The controls had no history of stroke, in either themselves or their families (Table 1).

### Data Pre-Processing

The probe-level data, in a CEL file format (Affymetrix Inc.), were converted into expression measures and background correction was performed via the robust multiarray average algorithm, set to default parameters, in the Bioconductor R ‘affy’ package. Three steps were involved in calculating the gene expression levels: (i) background correction; (ii) normalisation and (iii) expression value computation (12). If multiple probe sets corresponded to the same gene, the expression values of those probe sets were averaged. All the calculations were performed in RStudio, version 1.0.143, on the Windows platform.

### Differential Gene Expression Analysis

Differential Gene Expressions (DGEs) were obtained by applying a paired, two-tailed *t*-test to the calculated expression data. The gene expressions with a *P*-value < 0.01 were considered significantly altered genes. DEGs were also mapped with the DisGeNET (V3) database to determine gene-disease association.

### Gene Set Enrichment Analysis

GSEA was used for DEGs to define, a priori, the statistically significant gene sets showing concordant differences between IS patients and healthy controls. GSEA is a unique genomic tool that identifies significant subsets of genes expressed at the top and bottom of the gene rank list in a particular biological pathway (13).

## Results

### Differential Gene Expression Analysis in IS

**Figure 1:**
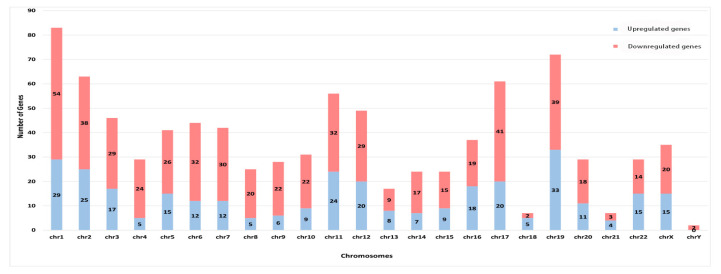
Chromosome wise count of DEGs in IS patients Note: Pairwise *t*-test was applied to screen DEGs (*P* < 0.01)

A total of 881 genes were found to be significantly altered in the stroke patients, where 324 were upregulated and 557 were downregulated. The highest numbers of deregulated genes in IS patients were observed on chromosome 1 (*n* = 83), followed by chromosome 19 (*n* = 72) (Figure 1). Chromosome Y did not show any upregulated genes.

### Functional Enrichment Analysis of Genomic Datasets in IS Using GSEA

**Figure 2:**
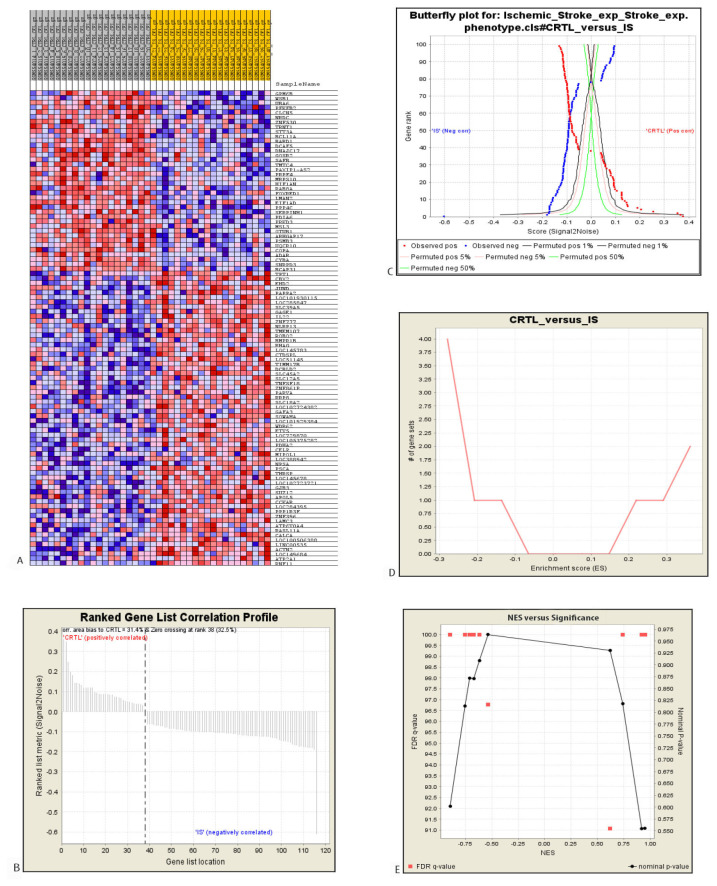
**A**. Heat map of the significantly altered genes in controls versus IS subjects. **B**. Ranked gene correlation profile of the enriched genes. **C**. Butterfly plot showing the pattern of gene expression between the controls and IS group. **D**. Global enrichment score histogram. **E**. NES (Normalised enrichment score) versus *P*-value

**Figure 3:**
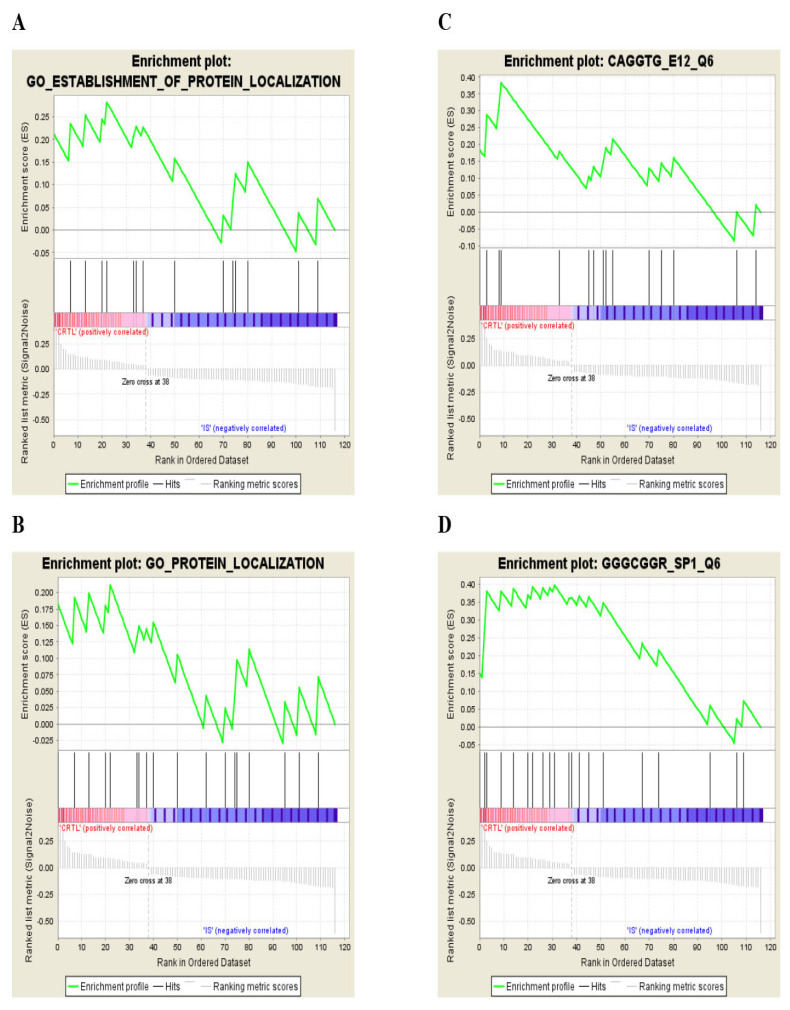
Enrichment plot of the respective GO annotations carrying over-represented genes. **A**. GO: Establishment of protein localisation. **B**. GO: Protein localisation. **C**. Enrichment plot of E12 (Tcf3) transcription factor targeting motif in the gene set. **D**. Enrichment plot of sp1 transcription factor targeting motif in the gene set. GSEA results are based on the Kolmogorov-Smirnov (K-S) statistical test

**Figure 4:**
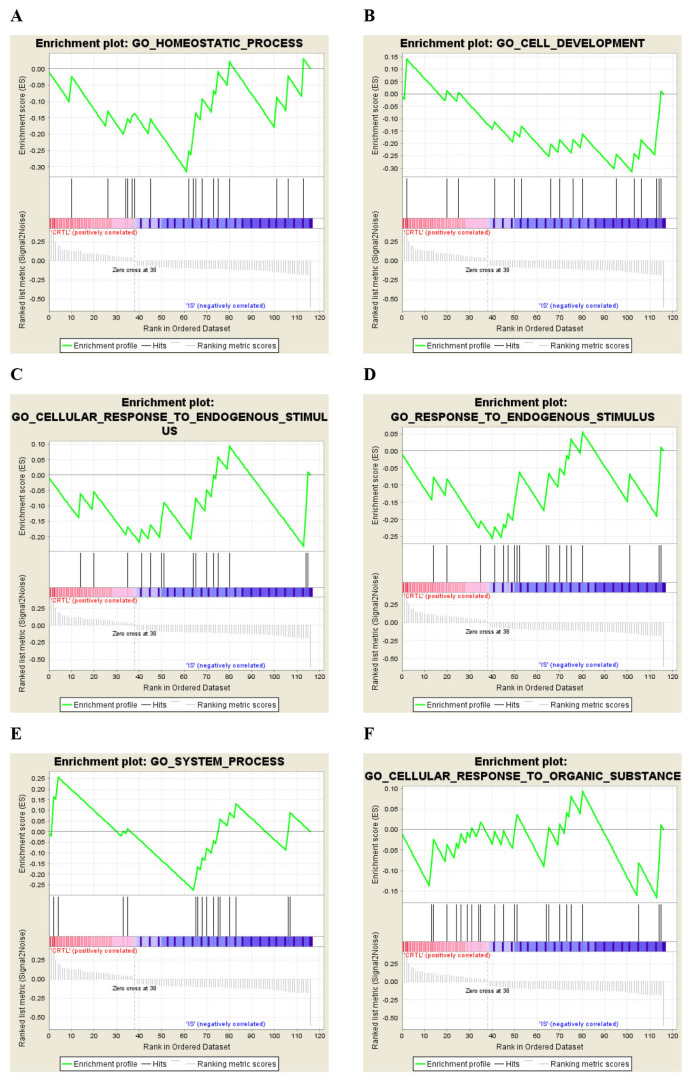
Enrichment plot of the respective GO annotations carrying under-represented genes. **A**. GO: Homeostatic process. **B**. GO: Cell development. **C**. GO: Cellular response to endogenous stimuli. **D**. GO: Response to endogenous stimuli. **E**. GO: System process. **F**. GO: Cellular response to organic substances. GSEA results are based on the Kolmogorov-Smirnov (K-S) statistical test

The gene sets were enriched via GSEA to study their functional significance related to specific molecular pathways. The identified datasets were core enriched to specific gene sets of molecular pathways or motifs (Tables 2–4). Two motifs and two GO processes incorporated core enriched, over-represented genes, which positively correlated with control datasets, as compared to IS (Table 2). Here, motif means the gene sets carrying potential transcription factor (TF) binding sites (GGGCGGR_SP1_Q6, CAGGTG_E12_Q6). In this case, SP1 and E12 are TFs (www.gsea-msigdb.org/gsea/msigdb/collection_details.jsp#C3). IS patients exhibited under-represented genes in the dataset enriched in 6 different GO annotations (Table 3). Significantly altered genes were segregated by GSEA in controls versus IS patients. The top 10 gene sets (with a minimum size of 15 genes and a maximum of 500) were filtered out of a primary gene set containing 17,786 genes. A total of 117 genes were filtered from the primary gene set and were subsequently arranged in a rank list, demonstrating significant enrichment score (ES) variations between controls and IS patients (Figure 2A). Thirty-eight marker genes (correlation area of 31.4%) were associated with controls and 79 marker genes (correlation area of 68.6%) were associated with IS patients (Figure 2B). The enrichment analysis (Figures 3A–3D) in the control phenotype defined 4 over-represented gene sets (out of 10), as shown in Table 2. In the IS phenotype, 6 gene sets were under-represented (Table 3) as shown in their enrichment results (Figures 4A–4F). A butterfly plot of the altered genes in the rank list graphically illustrates their correlation between the control and IS phenotypes (Figure 2C). The ES and normalised enrichment score (NES) histograms for the datasets were obtained via GSEA (Figures 2D and 2E).

The core enriched over-represented or under-represented genes were segregated in the respective GO annotated dataset (Tables 2 and 3). Table 4 summarises all the GSEA results for the control and IS phenotypes, revealing four over-represented and six under-represented gene sets annotated to specific GOs. The size of the gene sets varied from a minimum of 15 to a maximum of 22 genes. The highest and lowest ES and NES values among the over-represented gene sets was observed in those annotated to the TF motifs GGGCGGR_SP1_Q6 and GO_PROTEIN_LOCALISATION, respectively. The lowest ES and NES values among the under-represented gene sets were observed for the GO_HOMEOSTATIC_PROCESS TF motif.

Among the over-represented genes, the GO annotation CAGGTG_E12_Q6 had the best overall rank at maximum, characterised by the lowest nominal *P*-value (NOM *P*-value = 0.555), with a comparable false discovery rate *q*-value (FDR *q*-value = 1.000) and a familywise error rate *P*-value (FWER *P*-value) of 0.810 (Table 4). GO annotation GGGCGGR_SP1_Q6, however, had the lowest FWER (0.790) (Table 4). Among the under-represented genes, GO_HOMEOSTATIC_PROCESS was associated with the lowest NOM *P*-value (0.602) and FWER *P*-value (0.830), while the FDR *q*-value measures were comparable throughout the gene set. Among all gene sets, the highest leading edge signal was associated with GO_SYSTEM_PROCESS and GO_RESPONSE_TO_ENDOGENOUS_STIMULUS, which had high rankings of 53 and 77, respectively, suggesting that the maximum number of genes in the gene set were enriched (Table 4).

## Discussion

A review of extant literature examining genome-wide differential mRNA (gene) expressions and their association with known genetic defects in stroke or neurological disorders suggests that deregulated genes have characteristic relationships with either altered neuroaxonal physiology or pro-neurodegenerative metabolic events. The present research found 324 upregulated and 557 downregulated genes (*P* < 0.01), according to chromosome. Certain molecules expressed in the central nervous system (CNS) provide enhanced protection during toxic/stressful conditions, such as oxygen–glucose deprivation and excessive exposure to excitatory neurotransmitters (14). Kinesin family member 1B (KIF1B), located on chromosome 1p36.2, is a motor protein encoding gene required for transporting mitochondria into the neurons (15). KIF1B mutations result in non-demyelinating peripheral neuropathy, indicating that KIF1B is essential for neuronal function. Similarly, cytochrome c oxidase subunit Vb (COX5B) is essential for cellular metabolism and its over-expression in neurons also protects them from stressful conditions (14). High expression of KIF1B and COX5B in IS patients could potentially demonstrate the genes’ protective neurophysiological functions.

Dimethylarginine dimethylamino-hydrolase-2 (DDAH2) metabolises the toxic blood vessel inflammatory metabolite asymmetric omega-NG, NG-dimethylarginine (ADMA), which is also a risk factor for IS (16–17). Genetic alterations in DDAH2 are linked to haemorrhagic stroke, as has been observed in independent case-control studies (18). Another gene that regulates platelet and vascular homeostasis is UBASH3B (also known as TULA*-*2) and its low expression is associated with increased risk for thromboembolism in susceptible individuals (19). Ong et al. (20) have observed that UBASH3B is upregulated by 4.7-fold in hypertensive and hypercholesteraemic rabbits compared to sham controls. Stroke ischaemia enhances the rapid generation of reactive oxygen species (ROS), which damages cellular components and results in autophagy and necrosis (21). A similar outburst of ROS has been observed when reperfusion is facilitated following treatment (21). Glutathione plays a significant role in neutralising ROS generated in neurons after ischaemia (22). Glutaredoxin (GLRX3) also primarily participates in neutralising ROS species (23). Considering the significance of DDAH2, UBASH3B and GLRX3 to vascular and metabolic homeostasis, a substantial deregulation in their expressions can potentially increase IS risk.

Certain gene alterations result in SAM and HD domain containing deoxynucleoside triphosphate triphosphohydrolase 1 (SAMHD1) encoding deoxyribonucleotide triphosphohydrolase, which hydrolyses deoxyribonucleotide triphosphate (dNTP) (24). SAMHD1 mutations result in atherosclerotic changes in major vessels, predisposing people to early stroke (25–26). The activated peroxisome proliferator agonist receptor alpha (PPARA) gene enhances lipid uptake, as well as the use and upregulation of genes for beta oxidation (27–28). Administration of fenofibrate (a PPAR alpha agonist) in a rat IS model has reduced the infarct size, predominantly in male subjects (29). TP3 primarily regulates the cell cycle, apoptosis and oncogenesis. It is known to produce neuronal apoptosis in regions of cellular stress due to regional cerebral ischaemia. However, neuronal preconditioning due to a prior transient ischaemic attack (TIA) can enhance the expression of MDM2, which provides neuroprotection by antagonising TP53 (30). Thus, most of the deregulated genes found in this research corroborate with IS pathophysiology.

Deregulated genes have been landmark discoveries in the study of various human diseases. However, recent literature has established that, generally, it is not individual genes, but, rather, groups of genes (i.e. gene sets) and their products that cumulatively determine health phenotypes, disease conditions and therapeutic responses. Thus, analysing the association of genes towards a developing phenotype would be a more reliable and robust method than choosing individual genes and claiming that they alone expressed those characteristics. Along these lines, the current researchers used GSEA with DEGs to provide details of all such gene sets associated with IS (13).

The researchers filtered out 10 gene sets (minimum size of 15 genes, maximum size of 500), containing 117 genes, which were then ranked according to their ESs (Figure 2). Out these 10 gene sets, 4 contained over-represented genes and 6 contained under-represented genes. Interestingly, in 2 over-represented gene sets, 2 motifs determined the binding sites of the TFs (gene sets GGGCGGR_SP1_Q6 and CAGGTG_E12_Q6). GGGCGGR_SP1_Q6 (Molecular Signature Database) involved representative genes comprising the GC rich motif M6 GGGCGGR in the promoter region TF binding site V$SP1_Q6 (v7.4 TRANSFAC), which was identified by TF SP1 (Figure 3D). Among the over-represented genes, motif GGGCGGR_SP1_Q6 had the highest NES (Table 4). TF SP1 uniquely interacts with genes associated with regulating cell growth, differentiation, metabolism and apoptosis (31). In an experimental rat IS model, curcumin-mediated SP1 induction has improved neurophysiological functional recovery (32). In the present research, another gene set, CAGGTG_E12_Q6, was enriched with genes containing M12 CAGGTG promoter motif binding TF Tcf3 (Figures 3C). Tcf3 is involved in conserving neuronal progenitor cells (NPCs) during neuro-cortical development by suppressing wnt/beta-catenin signalling (33). Neuronal membrane glycoprotein M6-B (GPM6B), PFKFB2 and BCL11A were commonly enriched in both motifs targeting TF gene sets. GPM6B conducts housekeeping activities and is involved in neural development, cytoskeletal localisation and osteoblast differentiation (34). In the brain glycoprotein GPM6B is mostly concentrated in glial tissue or nestin-expressing neural stem cells and in the reactive astrocytes within injury penumbra (35). In this study, GPM6B was over-represented and enriched along with TRNT1, GOSR2, RAB8A and LMAN2 in two other GO biological processes based on protein localisation and its establishment subsets (Table 2; Figures 3A–3D). An earlier study has shown that neuronal subcellular protein localisation plays a major role in differential cerebral oedema following IS (36). GOSR2/Membrin and RAB8A encode trans-Golgi trafficking proteins to establish neural synaptic integrity and neurotransmission (37). Thus, in contrast to IS samples, their over-representation in controls suggests that these genes may have essential, protective neurophysiological effects.

This study observed under-expression of genes annotated to six biological processes that negatively correlated with IS (Figure 2B; Table 3). Restoration of homeostatic mechanisms has a vital effect on the prognosis and clinical outcomes of stroke (38).

Fundamentally, GSEA focuses on changes in the expressions of multiple genes in a coordinated way — as a group rather than as individual genes. Thus, GSEA can identify pathways whose several genes each change a small amount. This approach helps reflect many of the complexities of co-regulation and modular expression. GSEA uses a previously reported collection of annotated gene sets curated from various sources, including online pathway databases and extant biomedical literature. It also considers experiments with genome-wide expression profiles of samples belonging to IS and control groups. In this case, the genes were ranked according to the correlation between their expressions and the sample category distinction. The ‘size’ of the gene sets in Table 4 shows the number of differentially expressed genes (DEGs) in each set after filtering out all genes not in the expression dataset. The largest sets were GGGCGGR_SP1_Q6 (*n* = 20) and GO_CELLULAR_RESPONSE_TO_ORGANIC_SUBSTANCE (*n* = 22) in the over- and under-represented gene sets, respectively (Table 4).

The genes were positioned in the ranked list based on the maximum ES, highlighting gene sets with either a very large ES value (top ranked list FOR GO PATHWAY, Table 4) or a very small ES value (bottom ranked list FOR GO PATHWAY, Table 4). Information about the leading edge in the gene set was also found by calculating three measures: the tag (i.e. the number of leading edge subset genes that actively contribute to the ES), the list (i.e. the position or rank of the genes) and the signal (i.e. the strength/intensity of the genes). The leading edge consists of the genes that contribute the most to the gene set’s ES. The leading edge analysis is determined via that ES, which is defined as the maximum deviation from zero. The higher the score in the leading edge subset, the more relevant the gene set will be to disease pathophysiology. This research determined that the leading edge subsets with the highest values belonged to GGGCGGR_SP1_Q6 and to GO_RESPONSE_TO_ENDOGENOUS_STIMULUS in the over- and under-represented gene sets, respectively (Table 1).

The association between gene expression and the IS phenotype was depicted by the ES. The NES is an ES measure for an entire gene database, after normalisation across analysed individual gene sets. In this way, NES controls false positives by calculating the FDR corresponding to the ES. The current researchers calculated the NES by comparing observed and null distributions. NES scores from 0.62 to 0.95 and of −0.54 to −0.89 were obtained for the over-represented and under-represented gene sets, respectively. The NOM *P*-value represents the statistical significance of the ES. It depends on gene set size and is a highly stringent criterion, but it is limited because it cannot be adjusted according to gene set size. To determine the estimated probability of false positive findings, the FWER *P*-value and the FDR *q*-value were also computed. As they are stringent criteria, they were also not adjusted according to gene set size. Therefore, insignificant values may have occurred, as depicted in Table 4, for both the over- and under-represented gene sets.

During the GO Homeostatic process, the authors observed 10 core enriched genes that were negatively correlated with IS (Table 3). The GO homeostatic process also exhibited the lowest NES value. Fibroblast growth factor receptor 4 (FGFR4) is a receptor tyrosine kinase associated with cell multiplication, differentiation and metabolic homeostasis. The rs351855G/A (Gly388Arg) polymorphism and homozygous rs351855AA genotypic variations in FGFR4 have previously been found to increase susceptibility to stroke (39). Another gene, NCX3 (SLC8A3) when upregulated, it reduced the impact of neurological damage in cerebral ischaemic models by restoring sodium/calcium homeostasis (40). Boscia et al. (41) have observed that NCX3 gene silencing results in reduced myelination due to defective oligodendrocyte differentiation and maturation. In this study, the researchers observed that both FGFR4 and NCX3 were under-represented in 4 GO annotations (Table 3). Another important biological process related to this topic is that endogenous stimuli produce biological responses within living systems that are necessary for the survival of those systems. Pre-, per- and post-ischaemic conditioning activates myriads of cellular neural networks, which potentially enhance neuroprotection during IS (42). The GO biological processes pertaining to endogenous stimuli in this study exhibited under-expression of the following genes in IS patients: JunD, SLC39A5, GDF5, IRS4, FGFR4, SLC8A3, ACTN2, CALCA, ATP6V0A4, CCKAR, BMPR1B and ROBO2 (Table 3). Diaz-Cañestro et al. (43) have performed an in vivo knockout of JunD (which belongs to the AP-1 class of TFs), resulting in increased infarct size, poor neural activity, increased reperfusion injury, generalised inflammation and increased interleukin (IL)-1β production. They have also noted that there is low serum JunD expression in acute IS patients (43). Growth differentiation factor 5 (GDF5), when administrated to the post-traumatic cerebral injury site in rat brains, demonstrates enhanced neuronal regeneration and confers dendritic rearrangement, resulting in functional improvement (44). The present study’s observations suggest that three under-expressed genes — GDF5, NCX3 and FGFR4 — may be associated with neuronal regeneration. Restoration of the altered gene expressions described in the GO biological processes may also induce clinical and functional improvement. This must be further evaluated experimentally and validated in the context of IS management.

Combining genome-wide differential mRNA expression with GO and pathway enrichment is useful for substantiating different pathological subtypes of IS, according to Chinese traditional practice (45). Similarly, DEGs along with GO and pathway analysis, have revealed characteristic genomic alterations in IS associated with gender differences (46). In the present research, differential mRNA expression showed key under-expressed genes, which could play a putative role in aggravating neuronal pathophysiology; their downregulation in IS may also affect clinical outcomes.

This GO and pathway enrichment study found GPM6B to be the most commonly over-represented gene in all 4 gene sets, suggesting that its over-expression in control subjects may have a protective role in preventing neuronal injury and hastening regeneration (Table 2). The GPM6B gene encodes a membrane trafficking protein related to brain development, and its dysfunction could possibly be linked to neuronal ceroid lipofuscinosis and Rett syndrome (47–48). On the contrary, the relative under-expression of GPM6B in IS could be linked to increased neuronal damage and neurodegenerative processes. This research also found a significant under-representation of JunD, NCX3 and FGFR4 annotated to GO biological processes, which may significantly impact stroke pathophysiology and subsequent clinical outcomes.

The GSEA tool reveals crucial, highly relevant overlaps in enriched genes between groups. This comparison can be extended to different levels/stages in any study where gene expression deregulation is expected. This collective analysis using GSEA, DEGs and leading edge subsets provided insight into the transcriptomic changes and gene sets reflected in the IS phenotype. Following in preclinical and clinical validation, these gene sets will be more useful as markers than conventional genetic profiling. Additionally, individuals susceptible to IS can be identified early, depending on their deregulated gene sets. Though highly useful, these observations must still be affirmed or validated in preclinical and clinical setups in vitro, which is a limitation of in silico analyses.

## Conclusion

The current authors’ differential gene expression analyses showed that significantly altered genes have a pathophysiological relationship with neuronal dysfunction (as in neuropathies), elevated oxidative stress, hypoactive metabolism, poor toxin clearance and enhanced predispositions to IS at a young age. GSEA core enrichment also illustrated that particular gene sets are either under-represented or over-represented to specific GO annotations. GPM6B, which encodes a key membrane trafficking protein and is required for neuronal housekeeping functions and development, was over-represented in controls compared to IS patients. Under-representation of JunD, NCX3 and FGFR4 in IS can affect neuronal pathways related to homeostasis, development, response to internal or external cues and system processes. Thus, the current differential gene expression and enrichment analyses identified potential blood marker gene sets, which can predict IS development and clinical outcomes. However, future experiments will be required to validate these in-silico findings via various molecular approaches.

## Figures and Tables

**Table 1:** Clinical and demographical data of patients (GSE22255)

Group	Gender (male/female)	Mean age at examination (years)	*Age at onset (years)
Controls (*n* = 20)	10/10	58.7 ± 11.0	NA
Patients (*n* = 20)	10/10	60.2 ± 10.6	57.4 ± 10.8

Notes:

* Patients had only one stroke episode, at least 6 months before the blood collection and controls did not have a family history of stroke

**Table 2:** Core enriched over-represented genes in control subjects annotated to specific pathway

GO annotation	Over-represented genes
GGGCGGR_SP1_Q6	GPM6B, UBA6, PFKFB2, BCL11A, SAFB, RAB8A, LMAN2, PDIA6, OTUB1, PSMB3
CAGGTG_E12_Q6	GPM6B, PFKFB2, STT3A, BCL11A
GO_ESTABLISHMENT_OF_PROTEIN_LOCALIZATION	GPM6B, TRNT1, GOSR2, RAB8A, LMAN2
GO_PROTEIN_LOCALIZATION	GPM6B, TRNT1, GOSR2, RAB8A, LMAN2

**Table 3:** Core enriched under-represented genes in IS annotated to specific pathway

GO annotation	Under-represented genes
GO_HOMEOSTATIC_PROCESS	TEX15, FGFR4, SLC8A3, ATP2A1, CALCA, ATP6V0A4, CCKAR, SLC18A2, SLC12A5, RHAG
GO_CELL_DEVELOPMENT	ETV5, PARVA, SLC12A5, RHAG, BMPR1B, ROBO2
GO_SYSTEM_PROCESS	UBA6, CLCN5
GO_RESPONSE_TO_ENDOGENOUS_STIMULUS	JUND, SLC39A5, IL22, GDF5, IRS4, CA9, FGFR4, SLC8A3, ACTN2, CALCA, ATP6V0A4, CCKAR, SLC18A2, BMPR1B, ROBO2
GO_CELLULAR_RESPONSE_TO_ENDOGENOUS_STIMULUS	JUND, SLC39A5, GDF5, IRS4, FGFR4, SLC8A3, ACTN2, CALCA, ATP6V0A4, CCKAR, BMPR1B, ROBO2
GO_CELLULAR_RESPONSE_TO_ORGANIC_SUBSTANCE	GOSR2, SAFB, RAB8A, PPP4C, PDIA6, OTUB1, PSMB3, ADAR, CYBA, JUND, SLC39A5, GDF5, IRS4, FGFR4, SLC8A3, ACTN2, CALCA, ATP6V0A4, CCKAR

**Table 4:** Summarises the GSEA result of control phenotypes associated with over-represented dataset and IS phenotypes associated with under-represented dataset

Gene sets	Size	ES	NES	NOM *P*-value	FDR *q*-value	FWER *P*-value	Rank at max	Leading edge		
								Tags	List	Signal
Over-represented dataset										
GGGCGGR_SP1_Q6	20	0.40	0.95	0.556	1.000	0.790	31	50%	26%	56%
CAGGTG_E12_Q6	15	0.38	0.92	0.555	1.000	0.810	9	27%	8%	25%
GO_ESTABLISHMENT_OF_PROTEIN_LOCALIZATION	15	0.28	0.74	0.818	1.000	0.905	22	33%	19%	36%
GO_PROTEIN_LOCALIZATION	18	0.21	0.62	0.930	0.911	0.943	22	28%	19%	29%
Under-represented dataset										
GO_HOMEOSTATIC_PROCESS	17	−0.31	−0.89	0.602	1.000	0.830	56	59%	48%	96%
GO_CELL_DEVELOPMENT	16	−0.31	−0.75	0.813	1.000	0.906	15	31%	13%	31%
GO_SYSTEM_PROCESS	15	−0.27	−0.71	0.872	1.000	0.927	53	73%	45%	117%
GO_RESPONSE_TO_ENDOGENOUS_STIMULUS	18	−0.25	−0.67	0.871	1.000	0.943	77	83%	66%	206%
GO_CELLULAR_RESPONSE_TO_ENDOGENOUS_STIMULUS	15	−0.23	−0.62	0.909	1.000	0.948	4	13%	3%	12%
GO_CELLULAR_RESPONSE_TO_ORGANIC_SUBSTANCE	22	−0.17	−0.54	0.964	0.968	0.956	4	9%	3%	8%

Notes: MSigDB = molecular signatures database; ES = enrichment score; NES = normalised enrichment score; NOM *P*-value = nominal *P*-value; FDR *q*-value = false detection rate; FWER *P*-val = family-wise error rate
